# Low sensitivity of a urine LAM-ELISA in the diagnosis of pulmonary tuberculosis

**DOI:** 10.1186/1471-2334-9-141

**Published:** 2009-08-28

**Authors:** Klaus Reither, Elmar Saathoff, Jutta Jung, Lilian T Minja, Inge Kroidl, Eiman Saad, Jim F Huggett, Elias N Ntinginya, Lucas Maganga, Leonard Maboko, Michael Hoelscher

**Affiliations:** 1 Department of Infectious Diseases and Tropical Medicine, Klinikum of the Ludwig-Maximilians-University of Munich, Munich, Germany; 2 NIMR-Mbeya Medical Research Programme (MMRP), Mbeya, United Republic of Tanzania; 3 Centre for Infectious Diseases and International Health, Windeyer Institute for Medical Sciences, University College London, London, UK

## Abstract

**Background:**

The development and evaluation of rapid and accurate new diagnostic tools is essential to improve tuberculosis (TB) control in developing countries. In a previous study, the first release of a urine LAM-ELISA by Chemogen (Portland, USA) has been evaluated with a promising sensitivity and specificity for the diagnosis of pulmonary TB. In the present study, the now commercially available assay has been clinically assessed regarding its diagnostic value alone and in combination with clinical co-factors.

**Methods:**

The test was applied to two urine samples from 291 consecutively enrolled Tanzanian patients with suspected pulmonary tuberculosis. The participants were subsequently assigned to classification groups according to microbiological, clinical and radiological findings at recruitment and during a maximum follow up period of 56 days.

**Results:**

Only 35 out of 69 pulmonary TB cases -confirmed by smear microscopy and/or solid culture and/or liquid culture- showed at least one positive LAM-ELISA result (sensitivity 50.7%). The sensitivity was noticeably higher in females (66.7%) and in HIV positive participants (62.0%). The specificity amounted to 87.8% and was determined in participants with negative results in all microbiological tests and with sustained recovery under antibiotic treatment at day 56. Correlation with urinalysis revealed that proteinuria was significantly and positively associated with LAM-positivity (*P *= 0.026).

**Conclusion:**

This commercially available generation of LAM-ELISA does not appear to be useful as an independent diagnostic test for pulmonary tuberculosis. The question whether the assay is suitable as a supplemental device in the diagnosis of HIV-associated TB, requires further investigations.

## Background

Tuberculosis (TB) remains a serious global public health problem, primarily in developing countries affected by the HIV epidemic. Globally, 9.2 million new cases and 1.6 million deaths caused by tuberculosis (TB) were estimated only for the year 2006 [1]. An efficient TB control programme requires early and accurate diagnosis for screening, confirmation and the subsequent initiation of treatment. At present, the diagnosis of an active mycobacterial infection in low-income areas relies mainly on clinical examination, radiological findings, and identification of acid-fast bacilli in unprocessed sputum using a conventional light microscope. The sensitivity of sputum microscopy to identify active pulmonary TB is low since more than 10,000 bacilli per ml sputum are needed for reliable detection. Thus, smear-negative pulmonary TB is a common problem, especially in HIV infected individuals. Various studies classified 24% to 61% of HIV positive tuberculosis patients as smear-negative pulmonary TB [2]. Mycobacterial culture, which is regarded as the diagnostic gold standard, needs 10–100 viable bacilli per ml sputum and is therefore much more sensitive but requires a maximum incubation time of 6–8 weeks [3]. However, in resource constrained settings, culturing is still not widespread, because it demands expensive equipment, sophisticated setups and technical expertise [4]. Consequently, there is an urgent need for rapid, field adapted, inexpensive and accurate tuberculosis diagnostic tools.

In the last decades, the detection of mycobacterial antigens to diagnose tuberculosis has been subject of various research activities [5-10]. The diagnostic value of antigen detection per se has already been proven in a number of diseases, including malaria, influenza and bacterial meningitis. For the diagnosis of tuberculosis, special attention has been paid to lipoarabinomannan (LAM), a mycobacterium-specific lipopolysaccharide component of the bacilli's cell wall. In active mycobacterial disease, LAM is released into the blood and passes the renal barrier without major changes [11]. LAM is immunogenic and suspected to be an important virulence factor and therefore a potential drug target [12,13]. Various tests have been developed to detect LAM in serum [14,15], pleural effusion [16] or sputum [17-19] of tuberculosis patients. None of the tests is, however, widely used.

So far, the most promising approach was the detection of LAM in urine [20-24]. Urine can be easily obtained and its collection is often more culturally accepted than the collection of sputum or blood samples. In the last years, new assays using unprocessed urine [20] replaced a time-consuming approach requiring concentration and purification of the urine [21]. *Boehme et al*. assessed the first product generation of an ELISA for lipoarabinomannan in urine (MTB ELISA Test^®^, Chemogen, Portland, USA). The present study evaluated the commercially available assay produced by Chemogen. The test is now distributed as Clearview^® ^TB ELISA by Inverness Medical Innovations, Inc., Waltham, USA. The test was applied to urine from patients with symptoms of pulmonary TB to determine its diagnostic value alone and in combination with diagnostic co-factors.

## Methods

### Study site and population

The study was performed at the NIMR-Mbeya Medical Research Programme (MMRP) in collaboration with the Regional TB and Leprosy Co-ordinator and the Mbeya Referral Hospital. Situated in Southwest Tanzania, Mbeya Region has a total population of approximately two million. The region has a high burden of TB and HIV with 3601 TB cases notified in 2006 [25]. According to sentinel surveillance [26] and confirmed by yet unpublished data from a large population based cohort study the HIV prevalence in adults in the region is about 13% with a range from 8 to 19% at different sites.

300 adults with symptoms of pulmonary tuberculosis who had been referred from health facilities of Mbeya urban and rural districts were recruited at the TB clinic of the Mbeya Medical Research Programme between July and September 2007. The following inclusion criteria were used: persistent cough for ≥ 2 weeks and at least two other TB associated findings (haemoptysis, chest pain, fever, night sweats, malaise, recent unexplained weight loss, loss of appetite, contact with TB case), the ability to comply with study procedures such as sample collection and no TB treatment during the past 2 months.

### Study design

The recruitment of the study participants comprised interviews regarding medical history of participants, clinical examination, chest radiography, HIV pre-/ and post-test counselling, sample collection (3 or 4× sputum, 2× urine, 1× blood), HIV testing, urine dipstick testing (Multistix^® ^10 SG, Bayer Diagnostics, Bridgend, UK), sputum microscopy after Ziehl Neelsen staining, sputum culture on solid (Löwenstein Jensen) and liquid media (BACTEC™ MGIT, Becton Dickinson, Sparks, USA), and duplicate ELISA testing for lipoarabinomannan (MTB ELISA Test^®^, Chemogen, Portland, USA) in urine. Mycobacterial species were identified in AFB-positive culture material using Genotype^® ^Mycobacterium MTBC, CM and AS tests (Hain Lifescience, Nehren, Germany). The microbiology and the molecular biology laboratory of the Mbeya Medical Research Programme were operating according to standardised protocols and to quality control and assurance procedures.

Participants were assigned to well-defined classification groups according to microbiological and clinical findings at enrolment or during the 56 days of follow up. Patients eligible for TB treatment were treated by the District Leprosy and TB Co-ordinator following Tanzanian national guidelines. Patients diagnosed with HIV infection were referred for further staging and treatment to the Southern Highland Care and Treatment Programme which is supported by the Walter Reed HIV Program (funded by the Presidents Emergency Fund for AIDS Relief (PEPFAR)). Smear-negative patients, who did not require immediate TB treatment according to clinical symptoms, received two oral antibiotic regimens in order to treat possible other infections. The antibiotics comprised Amoxicillin and Co-trimoxazole, or alternatively Cefpodoxime (3^rd ^line medication).

### LAM-ELISA

The MTB-ELISA is a direct antigen sandwich immunoassay in a 96-well plate format. The blocked microtiter plates are pre-coated with purified LAM-specific antibodies. Participants' urines were first boiled at 95–100°C for 30 min and centrifuged for 15 min at 10000 rpm. From each sample two aliquots of 0.1 ml of the supernatant were applied to the same plate, incubated for 60 min at ambient temperature, and washed with Phosphate Buffered Saline pH 7.4/Tween-20 (PBST). Subsequently, 0.1 ml of undiluted conjugate solution (HRP-conjugated LAM-specific rabbit polyclonal antibody) was added. After 60 min incubation and washing with PBST, 0.1 ml of the colour developer (TMB) was administered to each well. The substrate was again incubated for 15 min at ambient temperature, and the reaction was stopped by adding 0.1 ml of stop solution (1 M H_2_SO_4_). The colour development was measured at 450 nm. One low positive and four negative controls were employed for each 96-well plate.

Each of the two urine samples that were both collected on day 1 of recruitment were divided into two aliquots for duplicate analysis. Results were regarded as valid if the mean optical density (OD) of the negative controls was less than 0.3. Samples were re-examined if the difference in OD between aliquots from the same sample was more than 15% of their mean value. If the mean OD of the aliquots was at least 0.1 above the mean OD of the negative controls, the sample was considered LAM positive. Patients were considered LAM positive if at least one of the two urine samples tested positive as described above.

### Statistical analysis

ELISA results were electronically retrieved from the analyser and stored in MS Access databases. Other study results were recorded on case-report forms, double entered into MS Access databases, compared and corrected for data entry errors. All statistical analyses were performed using Stata statistics software (version 10; Stata Corp., College Station, TX).

Diagnostic test performance (Sensitivity, specificity, predictive values and likelihood ratios) was calculated only in the groups with defined TB status (A&B as positives, C as negatives) using the "diagt" Stata component. The diagnostic likelihood ratio (DLR) [27] compares the probability of obtaining a correct test result with that of containing an incorrect test result for positive and negative test results respectively. Good tests should have a positive DLR that is well above unity and a negative DLR that is close to zero. DLR calculation uses the following formulas: positive DLR = sensitivity/(1-specificity); negative DLR = (1-sensitivity)/specificity.

The influence of HIV status, sex, CD4 count and urinalysis results on LAM positivity was assessed in multivariate models adjusted for several factors using Poisson regression with robust variance estimates [28,29].

### Ethical considerations

The study was approved by the Mbeya Ethics and Research Committee, Tanzania, and the National Ethical Committee/Medical Research Coordinating Committee, National Institute for Medical Research, Tanzania. The purpose and the procedures of the study were explained thoroughly to the attending TB suspects. Only persons who gave voluntarily written informed consent in the presence of a witness were enrolled in the study.

## Results

### Characteristics of the study population

The 291 study participants were categorized as follows: smear- and culture-positive for *M. tuberculosis *(group A; 16.5%); smear-negative, culture-positive for *M. tuberculosis *(group B; 7.2%); smear-negative, culture-positive for non-tuberculous mycobacteria (group B NTM; 15.5%); all smears and cultures negative and sustained recovery under antibiotic treatment at day 56 (group C; 28.2%); all cultures negative, chest x-ray and clinical symptoms very suspicious for TB (group D; 19.2%); any other possible combination of results and loss to follow up after recruitment (group I; 13.4%).

Although the number of female (151) and male participants (140) was similar in the total study population, group A had a higher proportion of men (64.6%) and group B a higher proportion of women (61.9%). The median age was 36 years (interquartile range = 30 to 46 years) and 172 (59.1%) patients were HIV infected (61.6% of females, 56.4% of males). The HIV prevalence in group A and B combined was significantly higher than in the remaining groups combined (Prevalence ratio = 1.319; *P *= 0.0098). 86.6% of the HIV infected participants had a blood CD4 cell count of less then 350 per ml and 58.1% had a CD4 cell count below 200 per ml blood. Interestingly, about 90% of the smear negative but culture positive individuals were HIV positive. The HIV positives in group A had higher CD4 counts than the HIV positives in group B (*P *= 0.374). The TB prevalence in HIV positive patients with defined TB status (groups A, B and C) was 57.5%, and 26.7% in HIV negative patients of the same groups. 18% of all HIV-infected patients (n = 291) had sputum smear-positive TB. Characteristics, findings at enrolment and medical history of the 291 participants are shown in Table 1.

**Table 1:** Patient characteristics, findings at enrolment and medical history

Characteristics		Findings at enrolment	% (n)	Symptoms during 3 months prior to enrolment	% (n)
Female	51.9%	HIV Infection	59.1 (172)	Chest pain	99.7 (290)
Mean age	38.8 years	*In HIV infected:*		Expectoration	96.6 (281)
Mean weight	53.9 kg	Blood CD4 count 0–199	58.1 (100)	Fever	81.4 (236)
Mean body temperature	36.7°C	Blood CD4 count 200–349	28.5 (49)	Body weakness	87.6 (255)
		Blood CD4 count ≥ 350	13.4 (23)	Night sweat	71.8 (209)
		Body temperature ≥ 37.5°C	16.6 (48)	Loss of appetite	49.5 (144)
		BCG Scar	66.1 (191)	Haemoptysis	11.7 (34)
		Lymphadenopathy	8.7 (25)	Oedema	3.1 (9)
		History of TB	12.0 (35)		
		TB contact	5.2 (15)		

### Diagnostic performance of the LAM-ELISA

If at least one of the two urine samples of the patient showed a positive LAM-ELISA result, the test was considered to indicate the presence of active tuberculosis infection in the per-patient analyses. In 89% of all patients both urine samples were either LAM positive or negative, only in 11% a disagreement of the two LAM-ELISA results was found. The overall sensitivity of the LAM-ELISA in patients with culture confirmed pulmonary *M. tuberculosis *infection (groups A and B) was 50.7%. Two major factors influenced the sensitivity considerably: sex and HIV status. The test sensitivity in women was 67%, while it was only 38% in men (*P *= 0.023). In HIV positive individuals the sensitivity was 62% compared to 21% in HIV negative participants. (*P *= 0.019) (Table 2). When combined in a multivariate Poisson regression model with LAM sensitivity as the outcome (true positives coded as "0", false negatives coded as "1"), the influence of HIV status and sex was less prominent than in separate univariate models. However, HIV status still had a significant association with LAM sensitivity (*P *= 0.040), and the association of sex with LAM sensitivity was still evident, although not significant anymore (*P *= 0.118) (Table 3). Further multivariate analyses showed that the influence of CD4 count adjusted for TB status, HIV status, sex and age on LAM-ELISA outcome was non-significant.

**Table 2:** Diagnostic test performance of LAM-ELISA (groups A and B were defined as gold standard positives, Group C as negative controls, other groups with undefined TB status were excluded)

		Sensitivity % (95% CI)	Specificity % (95% CI)	Positive predictive value % (95% CI)	Negative predictive value % (95% CI)	Positive diagnostic likelihood ratio (95% CI)	Negative diagnostic likelihood ratio (95% CI)
	**Subgroup (n)**						
**LAM positivity in at least one out of two urine samples**							
	**All** **(151)**	50.7 (38.4–63.0)	87.8 (78.7–94.0)	77.8 (62.9–88.8)	67.9 (58.2–76.7)	4.16 (2.23–7.78)	0.56 (0.44–0.72)
	**Females (79)**	66.7 (47.2–82.7)	83.7 (70.3–92.7)	71.4 (51.3–86.8)	80.4 (66.9–90.2)	4.08 (2.06–8.08)	0.40 (0.24–0.67)
	**Males** **(72)**	38.5 (23.4–55.4)	93.9 (79.8–99.3)	88.2 (63.6–98.5)	56.4 (42.3–69.7)	6.35 (1.56–25.80)	0.66 (0.50–0.85)
	**HIV-ve (64)**	21.1 (6.1–45.6)	91.1 (78.8–97.5)	50.0 (15.7–84.3)	73.2 (59.7–84.2)	2.37 (0.66–8.50)	0.87 (0.68–1.11)
	**HIV+ve (87)**	62.0 (47.2–75.3)	83.8 (68.0–93.8)	83.8 (68.0–93.8)	62.0 (47.2–75.3)	3.82 (1.78–8.21)	0.45 (0.31–0.66)

-ve = negative | +ve = positive

**Table 3:** Univariate and multivariate influence of HIV status and Sex on Lam Sensitivity

			Univariate Results			Multivariate Results		
**Covariate**	**Stratum**	**n**	**Prevalence ratio for specificity**	**(95% CI)**	**p-value**	**Prevalence ratio for specificity**	**(95% CI)**	**p-value**

HIV status								
	Negative	19	1			1		
	Positive	50	2.945	(1.193–7.272)	0.019	2.619	(1.045 – 6.561)	0.040
Sex								
	Female	30	1			1		
	Male	39	0.577	(0.359 – 0.927)	0.023	0.694	(0.440 – 1.097)	0.118

The results of a Poisson regression included only gold standard positive participants. The outcome variable (sensitivity) was coded as 1 for true positive LAM results and as 0 for false negative LAM results. Apart from age and sex no other predictors were included into the multivariate model.

Prevalence ratio for specificity = probability to receive a (correct) positive LAM result for TB infected patients who are HIV+ve or male, divided by probability to receive a (correct) positive LAM result for TB infected patients who are HIV-ve or female.

LAM-ELISA positive urine samples were found in 8.9% of the participants infected by non-tuberculous mycobacteria (B NTM) and in 12.2% of the non-TB patients (C). Therefore, the overall specificity amounts to 87.8%, being slightly higher in men (93.9%) and HIV negative participants (91.1%). The proportion of LAM positive patients in relation to their classification group and HIV status is shown in Table 4.

**Table 4:** Positivity of LAM-ELISA in at least one out of two urine samples

Classification	*Total* LAM +ve	*HIV -ve* LAM +ve	*HIV +ve* LAM +ve
**A (smear+ve/culture+ve, *M. tuberculosis*)**	27/48 (56.3%)	4/17 (23.5%)	23/31 (74.2%)
**B (smear-ve/culture+ve, *M. tuberculosis*)**	8/21 (38.1%)	0/2 (0.0%)	8/19 (42.1%)
**B NTM** **(smear-ve/culture+ve, non-tuberculous mycobacteria)**	4/45 (8.9%)	2/16 (12.5%)	2/29 (6.9%)
**C (controls, all smears and cultures-ve and sustained recovery under antibiotic treatment at day 56)**	10/82 (12.2%)	4/45 (8.9%)	6/37 (16.2%)
**D (all cultures-ve, CXR and clinical symptoms very suspicious for TB)**	9/56 (16.1%)	3/18 (16.7%)	6/38 (15.8%)
**I (any other possible combination of results and loss to follow up after recruitment)**	8/39 (20.5%)	4/21 (19.1%)	4/18 (22.2%)

+ve = positive | -ve = negative | CXR = chest x-ray

**Figure 1:**
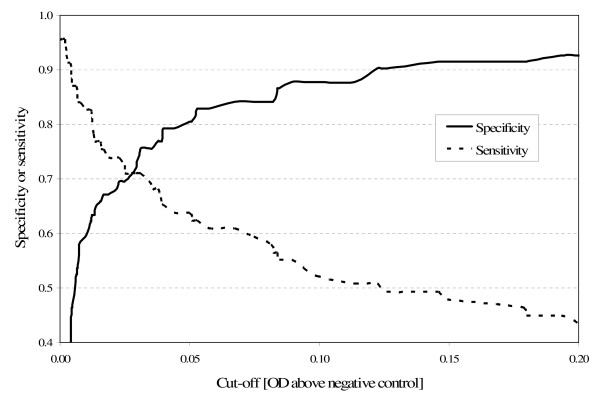
**Sensitivity and specificity of the LAM-ELISA at different cut-offs**.

We calculated the sensitivity and specificity of the LAM-ELISA at different OD cut-off values. Figure 1 illustrates the trade-offs that have to be made when trying to increase sensitivity or specificity by lowering or raising the cut-off.

The per-sample analysis of our results showed that only 44.2% of the urine specimens from microbiologically confirmed TB cases (A and B) were also LAM-ELISA positive, whereas 92.1% of the samples of non-TB cases (C) were LAM-ELISA negative.

### Association between performance of the LAM-ELISA and patient characteristics

Table 5 reflects the diagnostic performance of the LAM-ELISA in combination with various clinical findings or reported symptoms. The definitions of the symptoms reported by the patients are shown in Table 6. Combining LAM-ELISA results with patient characteristics or disease parameters did not substantially change the overall diagnostic value of the test. Improvements in sensitivity and positive predictive value were offset by impaired specificities and negative predictive values respectively. Only the combination of LAM-positivity and fever (body temperature ≥ 37.5°C) at the enrolment visit was different in this respect: this criterion improved test specificity from 87.8 to 98.8% and reduced the sensitivity by about 50% (from 50.7% to 23.9%). This is also reflected by a strong increase in the positive DLR (from 4.16 to 19.6) that is not totally offset by the increase in the negative DLR (0.56 to 0.77).

**Table 5:** Diagnostic test performance of LAM-ELISA combined with clinical parameters (groups A and B were defined as gold standard positives, Group C as negative controls, other groups with undefined TB status were excluded)

		Sensitivity % (95% CI)	Specificity % (95% CI)	Positive predictive value % (95% CI)	Negative predictive value % (95% CI)	Positive diagnostic likelihood ratio (95% CI)	Negative diagnostic likelihood ratio (95% CI)
	**Subgroup (n)**						
**LAM positivity in at least one out of two urine samples in combination with**							
**Lymphadenopathy**	**All (149)**	7.4 (2.4–16.3)	98.8 (93.3–100.0)	83.3 (35.9–99.6)	55.9 (47.4–64.2)	5.96 (0.71–49.80)	0.94 (0.87–1.01)
**Body temperature ≥ 37.5°C**	**All (149)**	23.9 (14.3–35.9)	98.8 (93.4–100.0)	94.1 (71.3–99.9)	61.4 (52.5–69.7)	19.60 (2.67–144.0)	0.77 (0.67–0.88)
Fever*	**All (150)**	44.9 (32.9–57.4)	91.4 (83.0–96.5)	81.6 (65.7–92.3)	66.1 (56.5–74.7)	5.20 (2.44–11.10)	0.60 (0.48–0.75)
Body weakness*	**All (151)**	49.3 (37.0–61.6)	89.0 (80.2–94.9)	79.1 (64.0–90.0)	67.6 (57.9–76.3)	4.49 (2.32–8.70)	0.57 (0.45–0.73)
Chest pain*	**All (151)**	50.7 (38.4–63.0)	87.8 (78.7–94.0)	77.8 (62.9–88.8)	67.9 (58.2–76.7)	4.16 (2.23–7.78)	0.56 (0.44–0.72)
Cough*	**All (151)**	50.7 (38.4–63.0)	87.8 (78.7–94.0)	77.8 (62.9–88.8)	67.9 (58.2–76.7)	4.16 (2.23–7.78)	0.56 (0.44–0.72)
Expectoration*	**All (151)**	47.8 (35.6–60.2)	87.8 (78.7–94.0)	76.7 (61.4–88.2)	66.7 (56.9–75.4)	3.92 (2.09–7.37)	0.59 (0.47–0.76)
Haemoptysis*	**All (151)**	2.9 (0.4–10.1)	98.8 (93.4–100.0)	66.7 (9.4–99.2)	54.7 (46.3–62.9)	2.38 (0.22–25.70)	0.98 (0.94–1.03)
Loss of Appetite*	**All (151)**	30.4 (19.9–42.7)	95.1 (88.0–98.7)	84.0 (63.9–95.5)	61.9 (52.8–70.4)	6.24 (2.25–17.30)	0.73 (0.62–0.86)
Night sweat*	**All (151)**	46.4 (34.3–58.8)	92.7 (84.8–97.3)	84.2 (68.7–94.0)	67.3 (57.8–75.8)	6.34 (2.82–14.30)	0.58 (0.46–0.73)

*Within 3 months prior to enrolment according to interviews regarding medical history

**Table 6:** Definition of symptoms reported by the patients

Symptoms during 3 months prior to enrolment	Definition
Cough	Acute or recurrent or persistent, non-remitting act of coughing
Chest pain	Discomfort or pain felt between the neck and the upper abdomen
Expectoration	Discharging mucus or other material from the respiratory tract by coughing
Fever	Subjectively or objectively (≥37.5°C) assessed rise of body temperature
Body weakness	Generalized lack of strength
Night sweat	Occurrence of excessive sweating at night, usually during sleep
Loss of appetite	Decreased appetite despite the body's basic caloric needs
Haemoptysis	Coughing up blood from the respiratory tract
Oedema	Swelling of the body due to fluid retention, predominantly in the lower legs and ankles

### Optical density values

**Figure 2:**
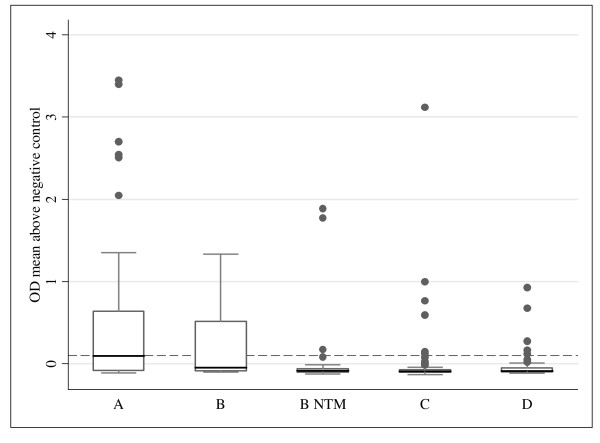
**Maximum optical density of the two samples by participant classification (median [line], interquartile range [box], upper and lower adjacent values [whiskers], and outside values [dots])**. N for each category is shown in Table 4; dashed line: cut-off at OD 0.1

The maximum OD of the two urine samples from each patient has been analysed for the classification groups and is shown in Figure 2. Apart from some outliers, maximum ODs above the cut-off were only reached in group A and B. However, even in group A the median of the maximum ODs (0.097) did not exceed the cut-off for test positivity (0.1).

### Urinalysis results and LAM-ELISA performance

The possible effect of urine parameters as determined by dipstick testing on LAM-ELISA positivity was examined in all samples with defined TB status (groups A, B, C; n = 302) using Poisson regression adjusted for potentially confounding factors (gender, HIV status, TB status and AFB smear-positivity). Of these parameters (protein, haemoglobin, leukocytes, nitrites, glucose, ketones, urinary pH, specific gravity, bilirubin, urobilirubin) only proteinuria had a significant influence on the LAM-positivity (Table 7).

**Table 7:** Influence of different urine parameters (according to dipstick testing) on positivity of LAM-ELISA adjusted for gender, HIV status, TB status and AFB smear-positivity (per sample analysis)

Finding	n	Prevalence ratio for LAM positivity	(95% CI)	p-value
**Protein**				
≤ Trace*	260	1		
> Trace	42	1.63	(1.06–2.52)	0.026
**Leukocytes**				
≤ Trace*	249	1		
> Trace	53	0.88	(0.54–1.43)	0.609
**Haemoglobin**				
≤ Trace*	241	1		
> Trace	61	1.48	(0.99–2.22)	0.057
**Specific gravity**				
≤ 1015*	164	1		
> 1015	138	1.16	(0.77–1.74)	0.484

*Baseline category for Poisson regression

## Discussion

Novel diagnostic approaches are urgently needed to improve TB diagnosis and TB control. Antigen detection methods for the diagnosis of TB have been developed as an alternative or a supplement to microscopy, growth-based detection, antibody tests, analysis of volatile organic compounds or other biomarkers, and to immunological assays. Detection of *M. tuberculosis *antigens especially in body fluids other than sputum has the following theoretical advantages: a) opportunity to quantify the organism load, b) possibility of high specificity, c) independence of a functioning immune response, d) applicability also in extrapulmonary TB [30]. Despite these promising characteristics in theory, none of the described antigen detection tests has yet been successfully employed in broader clinical practice. In the present study, a direct antigen-capture ELISA for mycobacterial lipoarabinomannan in boiled and centrifuged urine is comprehensively evaluated for its diagnostic value.

The sensitivity of the LAM-ELISA in culture confirmed TB patients of only 50.7% was disappointingly low. The specificity of 87.8% also fell far short of expectations. Moreover, the overall test performance can not substantially be improved by choosing a cut-off different from 0.1 (Figure 1).

The high expectations in the clinical value of the assay were mainly raised by a previous study of our group [20], performed in the same setting, which reported a much higher sensitivity (80.3%) and specificity (99%). This study by *Boehme et al*. recruited fewer female (151 f/140 m vs. 95 f/136 m; *P *= 0.014) and more HIV-infected (172 HIV+ve/119 HIV-ve vs. 147 HIV+ve/66 HIV-ve; *P *= 0.023) participants than the present trial. Nevertheless, these differences alone can not explain the discrepant results, since analyses in male and HIV-infected subgroups of both studies still result in a considerably lower sensitivity and specificity of the LAM-ELISA in our study.

Another difference between the two studies is that *Boehme et al*. used only results from conventional solid media (LJ) for overall case definitions whereas we used a combination of solid and liquid culturing methods, with its superior diagnostic precision [31]. However, our results for sensitivity and specificity still remain similar when liquid culture is not considered for case definitions (51.5% and 87.8% respectively).

The first published clinical evaluation of LAM-ELISA in urine by *Tessema et al*. [24], showed a higher sensitivity (81.3%) than our study, but a similar specificity (86.9%) while using a case definition requiring the presence of at least two AFB-positive sputum smears.

Data regarding the LAM-ELISA presented at the 16th Conference on Retroviruses and Opportunistic Infections 2009 by *Mutetwa et al*. [32] support our findings. This group reports an overall sensitivity of 44% and an overall specificity of 89%.

Unlike most of the previous LAM-ELISA studies, our study employed molecular biological differentiation between *M. tuberculosis *and non-tuberculous mycobacteria (NTM) which should actually improve the apparent sensitivity of the test by improving the specificity of the gold-standard. However, despite the use of more precise reference diagnostic, we observed a lower sensitivity instead.

The most likely explanation for the poor diagnostic performance of the test is, that the changes in the assay are accountable for the impaired sensitivity compared to previous investigations, particularly that of *Boehme et al*. who used a different version of the test by the same manufacturer. Unfortunately, we are unable to provide information what exactly are the differences between the two versions of the test. The manufacturer's information on reagents and test configuration only provides a broad picture on the principle of the test, but lacks important specifications such as the type of antibodies used for antigen capture.

Because a single-gate patient sampling strategy identifying TB cases and the Non-TB controls in the same source population was used to avoid spectrum bias [33], the presented specificity values should be considered more applicable to a real life situation than specificity data generated with healthy volunteers as the control group [20]. The real life situation is also obvious in the fact that most of the patients were suffering from several TB associated symptoms for more than 3 months prior to enrolment which indicates an advanced state of diseases.

In-vitro analysis has shown that the LAM-ELISA can detect both *M. tuberculosis *and non-tuberculous mycobacterial species, but the latter only at significantly higher concentrations [20]. At first sight it seems that the LAM-ELISA in our study determines mycobacterial infections other than tuberculosis in almost 9% of the patients. However, the fact that a similar percentage of controls are also LAM-ELISA positive suggests that the positive results in patients with solely NTM infection are not really attributable to cross-reactivity of the test.

The sensitivity of the LAM-ELISA is noticeably higher in HIV-infected patients (62%) than in HIV-negatives (21%); while the specificity in HIV-infected patients is lower (84% in HIV positive vs. 91% in HIV negative). Because the sensitivity of sputum microscopy and culture techniques in HIV infected persons with advanced immunodeficiency is low, it is be theoretically possible that false negative gold standard assessments could have led to a misclassification of some LAM-ELISA results and thus caused the low specificity of the assay. However, the influence of CD4 counts on LAM-ELISA results adjusted for TB status, HIV status, sex and age in a multivariate model was small and far from significant. Furthermore, the group of HIV+ve, LAM+ve TB-ve persons had relatively high CD4 counts when comparing them to HIV+ve LAM+ve TB+ve. Consequently, this is an unlikely explanation for the low specificity of the test.

A recently published study [34] showed that among TB cases lower CD4 cell counts are associated with a higher likelihood of a positive LAM-ELISA in urine, which would make the assay a promising rapid TB diagnostic tool among patients with advanced immunodeficiency. However, our data do not support this finding since CD4 counts were not significantly associated with the LAM-ELISA test outcome.

LAM-ELISA seems to diagnose TB infection in patients presenting with fever at enrolment with both high specificity and high positive DLR. Further investigations on LAM-ELISA have to establish if these findings can be generalised, helping to diagnose TB in febrile suspects. Combination with other clinical parameters did not substantially influence the overall diagnostic performance of the LAM-ELISA.

Among the analysed urine parameters, proteinuria had a significant positive association with LAM-positivity. Haematuria also had a positive association which was however only marginally significant. This kind of interference has not been reported yet.

In conclusion, the evaluated version of the antigen-capture LAM-ELISA does not seem to fulfil the requirements for a stand-alone diagnostic test for pulmonary tuberculosis.

In our opinion, further investigations are needed to elucidate if the LAM-ELISA, in this stage of development, is valuable as a supplemental tool for the diagnosis of HIV-associated TB. This seems particularly important, when taking into consideration that TB is one of the most important opportunistic infections of HIV patients and that the sensitivity of smear microscopy in immunocompromised patients is low [2,30,35]. The more encouraging earlier results for other versions of the test might indicate that changes in its setup could improve diagnostic performance considerably. It would therefore be highly desirable to further develop the LAM-ELISA into the field-adapted and precise diagnostic tool that is urgently needed for TB diagnosis and control in resource limited settings.

## Conclusion

The commercially available generation of urine LAM-ELISA does not appear to be useful as an independent diagnostic test for pulmonary tuberculosis.

## Competing interests

The authors declare that they have no competing interests.

## Authors' contributions

KR, ElS, JFH and MH designed the study. KR wrote the study protocol. KR, LTM, LuM and LeM were overseeing and/or conducting the recruitment, examination, and follow-up of the study participants. LTM was responsible for sample collection. JJ was in charge of TB microbiology and PCR laboratory work. IK supervised the LAM-ELISA. KR, ElS, ENN and EiS carried out data management, analysis and interpretation. KR wrote the manuscript with major contributions from ElS, MH, and the other authors. All authors read and approved the final manuscript.

## Pre-publication history

The pre-publication history for this paper can be accessed here:

http://www.biomedcentral.com/1471-2334/9/141/prepub
